# Preventing suicides: need for a stronger strategy

**DOI:** 10.1016/j.lansea.2023.100276

**Published:** 2023-09-08

**Authors:** The Lancet Regional Health – Southeast Asia

In August 2023, India was shocked when a teenage medical aspirant and his father died by suicide. In India, the suicide death rate (per 100,000 population) increased from 9.9 in 2017 to 12 in 2020. Considering its large population, India reports the largest number of suicides in the world with 164,033 cases reported in 2021. A highly effective suicide prevention strategy is therefore of the utmost importance.

Depression is one of the most common mental health conditions among people who die by suicide. However, the stigma associated with suicide usually discourages individuals with suicidal ideation from seeking help or confiding in others about their problems. Risk factors for suicide are sometimes not externally evident. Childhood trauma, unemployment, and being socially disadvantaged may contribute to an individual's predisposition or vulnerability to mental health issues. For example, childhood trauma might affect the DNA methylation and expression of the hippocampal glucocorticoid receptor gene, leading to more cortisol release (enhanced stress response) during adulthood. The first step towards the elimination of stigma associated with suicide is progression of the public’s opinion towards empathy for people experiencing suicidal behaviour.

Gender discrimination, gender-based violence, and early marriage lead to increased suicides among adolescents in India. Unemployed married women constituted 51.5% of the women who died by suicide in 2020. However, the pattern of deaths by suicide in India is changing. In their Comment in this issue, Yadav and colleagues discuss the increasing suicide death rates among men compared with women in India (2.5 times higher in 2021). Compared with 2014, the suicide death rates among daily wage workers increased by 170% in 2021. It is worrisome that the suicide death rate in married men was three times that of married women. Impulsivity, fearlessness about injuries, and alcohol or drug consumption, especially in men, can increase vulnerability to suicide. Family problems and reluctance to seek help must be resolved by continued follow-up and community support.

Social support is essential for building resilience in children and adolescents. High expectations from society (socially prescribed perfectionism) is a major risk factor for suicide in this age group, as they can lead to self-criticism and social disconnection. Parents, schools, and education departments must ensure that children receive education in psychologically safe environments, with ample time to play, long before they start to face the responsibilities of adulthood. There must also be more social-emotional learning programmes in addition to improving coping and problem-solving skills in children.

We discussed the importance of early intervention for mental wellbeing in our previous Editorial. People with depressive disorders must be identified early, including special consideration of individuals with a history of self-harm, suicidal behaviour, and less social connection. It is important to develop targeted interventions for phases of transition in life such as adolescence and motherhood. Consumption of pesticides and hanging are the most common methods of suicide in Southeast Asia. Pesticides and blankets are easily accessible which makes it difficult to prevent suicides via these methods. However, banning of pesticides is plausible and was highly successful in decreasing suicides in Sri Lanka. As alcohol brings revenue to the government (same as drugs in a few countries), restriction of addictive substances as part of a suicide prevention strategy is challenging and strongly depends on the commitment of politicians. Religion and spirituality can also help individuals, particularly during different crises in life such as bereavement. Onie and colleagues, in their Comment, call for the support of religious leaders in Indonesia for suicide prevention, with religion being a central part of Indonesia’s society. Joint statements from religious organisations would help in suicide prevention.

A healthy work environment and economic stability are critical factors for mental wellbeing. Labour departments are responsible for upholding the rights of workers and must try to prevent hostile work environments, considering the rise in suicides among medical professionals as well as defence personnel. Also, there must be more laws for the protection of workers. There was an increase in suicides after the economic crisis in Sri Lanka and loss of employment during the COVID-19 pandemic. Financial support from charitable organisations or government would help. However, ensuring that financial support really reaches those in need without red tape is another challenge.

In their recent review in *The Lancet Psychiatry*, O'Connor and colleagues discussed various measures to prevent deaths due to mental illness. All countries in the Southeast Asia region need strong policies to guide action on suicide prevention and save precious lives. Most importantly, all countries must ensure that the rights of people with mental health issues are the same as those of people with physical health issues. In India and Indonesia, there is a requirement for more robust surveillance and registration of injuries related to suicides at hospital and police station level. Further research is required on a regular basis to estimate the effectiveness of methods promoted by national suicide prevention strategies. In addition, people with lived and living experience of mental health issues, and family or friends who care for them, must be involved in the planning of suicide-related research. Suicides are preventable, and a combined effort is required at society and national levels. Lending an ear whenever possible is the first step.

